# Mediterranean Diet Compliance Is Related with Lower Prevalence of Perceived Stress and Poor Sleep Quality in University Students: A Cross-Sectional Study in Greece

**DOI:** 10.3390/nu17132174

**Published:** 2025-06-30

**Authors:** Antonios Dakanalis, Konstantinos Papadimitriou, Olga Alexatou, Georgia-Eirini Deligiannidou, Myrsini Pappa, Sousana K. Papadopoulou, Aikaterini Louka, Georgios Paschodimas, Maria Mentzelou, Constantinos Giaginis

**Affiliations:** 1 Department of Mental Health, Fondazione IRCSS San Gerardo dei Tintori, Via G.B. Pergolesi 33, 20900 Monza, Italy; antonios.dakanalis@unimib.it; 2 Department of Medicine and Surgery, University of Milano Bicocca, Via Cadore 38, 20900 Monza, Italy; 3 Department of Nutritional Sciences and Dietetics, School of Health Sciences, International Hellenic University, 57400 Thessaloniki, Macedonia, Greece; kpapadimitriou@ihu.gr (K.P.); deligiannidoueirini@yahoo.gr (G.-E.D.); souzpapa@gmail.com (S.K.P.); 4 Department of Food Science and Nutrition, School of Environment, University of Aegean, 81100 Myrina, Lemnos, Greece; fnsd23003@fns.aegean.gr (O.A.); fnsm23028@fns.aegean.gr (M.P.); loukathy612@gmail.com (A.L.); maria.mentzelou@hotmail.com (M.M.); 5 Department of Medicine, School of Health Sciences, Democritus University of Thrace, 68100 Alexandroupolis, Evros, Greece; 6 Department of Secondary Education of Preveza, Ministry of Education of Religions and Sports, Anthousa, 48060 Parga, Preveza, Greece; g.paschodimas@yahoo.gr

**Keywords:** Mediterranean diet, perceived stress, sleep quality, university students, sociodemographic factors, lifestyle factors, mental health, academic performance, quality of life, Greece

## Abstract

Background/Objectives: Mediterranean diet (MD) adherence is associated with a lower risk of developing several chronic diseases, including cardiovascular and metabolic disorders, cancer, and mental health diseases. University students are vulnerable to mental disorders that considerably affect their well-being and quality of life, which may be ascribed to the stressful environment during their academic studies. This cross-sectional survey designed to explore the relationship between MD compliance and perceived stress levels as well as sleep quality in a representative sample of university students from Greece. Methods: This survey assigned 5433 university students from 10 Greek regions. We used qualified questionnaires to examine the socio-demographics of the assigned students. Anthropometrics were determined by qualified methods. Validated questionnaires were used to determine diverse lifestyle factors like physical activity, perceived stress, sleep quality and MD adherence. Results: Concerning the primary objectives of this study, greater MD compliance was independently and significantly related to lower incidence of perceived stress and poor sleep quality in university students. As far as the secondary objectives of this study concern, greater MD compliance was independently and significantly related to female gender, rural type of residence, living with family, smoking, biomedicine studies, being normal weight, and having enhanced physical activity. Conclusions: This study reinforces the idea that healthy dietary patterns like MD could be a significant modifiable factor against perceived stress and poor sleep quality of university students. Based on this evidence, longitudinal studies should be performed to confirm whether MD can exert a causal effect on perceived stress and sleep quality.

## 1. Introduction

The Mediterranean Diet (MD) has gathered increasing attention in recent years for its broad health benefits. Finicelli et al. [1] reported a significant rise in clinical trials over the past decade, highlighting the MD’s positive effects on conditions like cardiovascular disease [2], metabolic disorders [3,4], gastrointestinal disorders [5], and cancer [6], largely due to its anti-inflammatory and antioxidant actions [7,8,9]. All the above beneficial effects may be attributed to the fact that MD includes an increased intake of whole grains, fruits and vegetables, and a low intake of red meat, sweets and beverages as well as ultra processed foodstuffs. From a mechanistic point of view, MD can mainly exert lipid-lowering effects as well as protective antioxidant, and ant-inflammation properties [7,8,9]. Moreover, MD can act against platelet aggregation, while it can also exert diverse modifications of hormones and growth factors that participate in tumor disease progression. It can also lead to suppression of nutrient recognizing paths by specific amino acid constraint, suppressing gut microbiome-facilitated production of metabolites, which influences metabolic health [7,8,9]. In addition, MD includes numerous phytochemicals, such as polyphenols, flavonoids, triterpenoids, etc., that are able to act against obesity and metabolic disorders [10,11,12]. Their anti-obesity actions contain the inhibition of adipocyte differentiation, browning of the white adipose tissue, and suppression of enzymes, including lipase and amylase. MD also leads to suppression of inflammatory environments, improvement of the gut microbiota, and reduction in obesity-related gene expression [10,11,12].

Meanwhile, Vélez-Toral et al. [13] explored the MD’s psychological influence, demonstrating a positive association between MD adherence and psychological well-being in university students, including higher self-self-confidence, life happiness, and perception of coherence. This connection is largely mediated by greater intake of healthy foodstuffs like fruits and vegetables and reduced consumption of unhealthy items [13]. Recently, certain surveys have suggested that university students’ nutritional behaviors are turning away from MD standards to less healthy nutritional behaviors, particularly for students living away from home and even for students with a Mediterranean country origin [14]. Greater MD compliance was related to lower likelihood of depression and anxiety, and greater levels of perceived stress with reduced fruit and vegetables consumption [14,15]. Other studies also suggested that implementing a dietary pattern like the MD may be related to improved emotional well-being and quality of life of university students [16,17]. A recent cross-sectional survey also reported that MD was positively related to mental well-being among university students [18].

Nowadays, psychological disorders such as perceived stress vary across different life stages, such as childhood, adolescence, adulthood, and elderly, influenced by developmental and environmental factors. Lutin et al. [19] found that children primarily interpret stress through emotional valence rather than arousal, with age and gender influencing arousal sensitivity, suggesting that stress perception in youth is complex and developmental. Johnson et al. [20] recorded stress from age 25 to 50, revealing a general decline over time, with lower and declining stress linked to better midlife psychological, physical, and relational well-being, emphasizing the long-term impact of stress trajectories. In older adults, Commodari and Di Nuovo [21] suggested that stress perception is strongly associated with life appraisal and living environment rather than objective health, advocating for holistic aging support that fosters autonomy and psychological well-being. Perceived stress has recently been considered as a potential risk factor which may considerably affect the daily quality of life as well as well-being and academic performance of university students [22,23,24,25,26].

An additional factor for overall health across all age groups is sleep quality [27]. For young adults, sleep quality is significantly affected by parameters like night eating syndrome, healthy dietary habits (such as MD) [28], sleep mood, pain experiences, and social network engagement, with these variables explaining 33% of the variance in sleep outcomes [27]. Sleep quality is also shaped by attributes like efficiency, duration, and wake time, and influenced by physiological and psychological factors [29]. Poor sleep quality was linked to harmful outcomes such as fatigue, irritability, and impaired daytime functioning [29]. In older adults, Koffel et al. [30] emphasized tailored interventions, recommending strategies focused on light exposure, physical activity, and structured routines to promote sleep health. A considerable factor that influences sleep quality is perceived stress in both children [31], and older adults [32], and college students [33]. Specifically, in college students, Huang et al. [33] found that stress negatively impacts sleep, and this effect was ascribed to lower presence of meaning in life and higher depression. Particularly, concerning students who searched for meaning, the harmful impacts of perceived stress and depression on sleep quality were weakened [33]. Simultaneously, the positive effect of presence of meaning in life on sleep quality was enhanced [33]. It should be speculated that the presence of meaning in life is a crucial psychological cognitive resource, denoting that people understand the meaning of their life and acknowledge their life’s purpose, objectives, and task [33]. The presence of meaning in life exerts a considerable impact in improving physical condition and mental health [33]. Moreover, a systematic review and meta-analysis suggested that psychological treatments may enhance sleep quality for young adults such as university students [34]. Another systematic review and meta-analysis provided evidence that there is an enhanced incidence of poor sleep quality amongst African university students [35]. Students with stress and being in the second year as well as those utilizing electronic devices at bedtime and suffering from chronic diseases showed considerably poor sleep quality [35]. Unhealthy dietary behaviors may also negatively influence the sleep quality of university students [36,37,38].

Together, the above findings reinforce the probable beneficial impacts of the MD against psychological disorders like perceived stress, as well as against poor sleep quality. Therefore, the present survey was designed to provide valuable insights into MD adherence and its potential associations with sociodemographic and lifestyle factors, including perceived stress and sleep quality amongst university students.

## 2. Methods

### 2.1. Study Population

**Figure 1:**
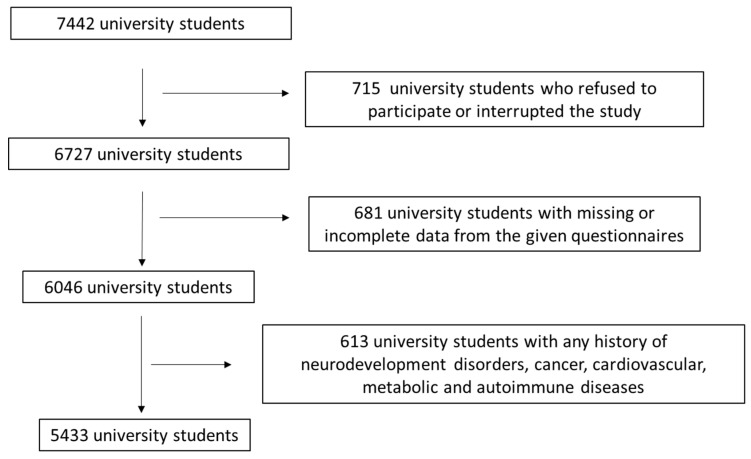
Flow chart diagram of the study population enrollment.

This is a cross-sectional survey initially contained 7442 university students recruited by 10 different, both rural and urban, areas from Greece (Athens, Thessaloniki, Larissa, Kalamata, Kavala, Korinthos, Alexandroupolis, Patra, South and North Aegean). It exclusively assigned active university students from the above 10 Greek regions. The enrollment to the study was from March 2021 to April 2025. Figure 1 illustrates the study population enrollment. By applying several exclusion and inclusion criteria (Figure 1), 5433 active university students were conclusively included, resulting in a final response proportion of 73.0%. An initial inclusion criterion was any active students aged from 18 to 25 years old, and a second inclusion criterion concerned students from Greek universities. The first exclusion criterion included any initially recruited active students who refused or interrupted the study at any stage. By applying the first exclusion criterion, 6827 students remained. The second exclusion criterion was any active students who had missing or incomplete data from the questionnaires given. By applying this exclusion criterion, 6046 students remained. The last exclusion criterion was any active students with any history of neurodevelopment disorders, cancer, cardiovascular, metabolic and autoimmune diseases. By using the 3rd exclusion criterion, 5433 students finally remained.

The Ethics Committee of the University of the Aegean permitted the current survey (ethics approve code: no 21/11.10.2017, approve date: 11/10/2017) in accordance with the World Health Organization (52nd WMA General Assembly, Edinburgh, Scotland, 2000). All the participants’ data were confidential, and none of the participants had any history of chronic diseases, like cardiovascular diseases, metabolic disorders, mental disorders, or tumor malignancies. In addition, all participants were informed concerning the goals of the current survey by signing a written informed consent. Sample size computation was conducted using PS: Power and Sample Size estimator software v2. A series of random binary numbers (i.e., 100100101, in which 0 showed recruitment and 1 not recruitment to the study) was utilized for the randomization process. The calculation of the power of the present sample size indicated a power of 88.8%.

### 2.2. Assessment of Sociodemographic and Anthropometric Factors

Sociodemographic parameters, like age, gender, nationality, residence type, family financial level, living status, parents’ marital status, smoking habits, and employment status, were collected by relevant questionnaires. Additionally, all students were assigned from Greek universities, and their academic performance as well as the type of their studies were documented. All the socio-demographic information was collected through face-to-face interviews between assigned university students and certified personnel to minimize recall biases. Family financial level was classified based on the yearly family income as low for family yearly income ≤10,000 €, medium for yearly income >10,000 € and ≤20,000 €, and high for yearly income >20,000 €. Academic performance was categorized according to the mean degree of courses finalized at the time of study as good for degree between 5.0 and 6.49, very good for degree between 6.5 and 8.49, and excellent for degree between 8.5 and 10.0 according to the official principles of the Minister of Education of Greece.

Body Mass Index (BMI) was calculated by measuring the body weight and the height of the assigned university students at the time of study. University students’ weight was measured by a Seca scale (Seca, Hanover, MD, USA), with no shoes, to the closest 100 g, and height was measured by a portable stadiometer (GIMA Stadiometer 27335, Gima Professional Medical Products, Athens, Greece) without shoes, to the closest 0.1 cm [39,40]. The WHO standards were applied for categorizing the assigned university students as normal weight, overweight or obese [39,40].

### 2.3. Evaluation and Lifestyle Factors

Physical activity levels were determined by the International Physical Activity Questionnaire (IPAQ). In this questionnaire, the study participants state the frequency of exercise they did in a regular week. IPAQ is utilized worldwide and determines the whole physical activity concerning the last seven days with the aim to classify it as low, moderate, or high [41]. IPAQ has been checked in both developed and developing countries and showed good consistency and acceptable validity. Briefly, the goal of IPAQ-Greek is to sum up vigorous, moderate and walking physical activities during the former seven-days period that leads to an overall physical activity score, expressed in MET-minutes per week (MET.min.wk-1) [41].

The levels of stress of the recruited individuals were assessed by the Perceived Stress Scale (PSS) [42]. This is a 10-item questionnaire, which aims to evaluate the self-reported amount of stress by assessing thoughts and feelings during the previous months. PSS showed great reliability and acceptable validity [43]. Each question takes a score from 0 (never) to 5 (very often). The whole score from the sum of responses can be ranged from 0 to 40 and the perceived stress is higher as the whole score increases. The PSS is classified to 3 groups, which are low perceived stress (low PSS; 0–13), medium (medium PSS; 14–26), and high perceived stress (high PSS; 27–40) [43].

Sleep quality was determined by the Pittsburgh Sleep Quality Index (PSQI) which contains 19 items that are rated on a four-point scale (0–3) and clustered into seven components (sleep quality, sleep latency, sleep duration, habitual sleep efficiency, sleep disturbance, use of sleeping medications, and daytime dysfunction) [44]. The item scores in every component were summed and transformed into component scores ranging from 0 (better) to 3 (worse) based on the guidelines [44]. The whole PSQI score was estimated as the summation of seven component scores ranging from zero to 21, where higher score shows worse condition. A whole PSQI score of <5 is indicative of adequate sleep quality guidelines [44].

MD compliance of the enrolled university students was determined by applying the KIDMED questionnaire [45]. The KIDMED questionnaire constitutes one of the most broadly used scoring systems to assess MD compliance of children, adolescents and young adults. This questionnaire shows great consistency and validity and includes 16 questions for determining dietary behaviors. Twelve questions get a positive score and four questions get a negative score. In fact, it contains 4 questions representing a negative effect to the MD compliance (consumption of fast food, baked goods, sweets, and skipping breakfast) and 12 questions representing a positive connotation (consumption of oil, fish, fruits, vegetables, cereals, nuts, pulses, pasta or rice, dairy products, and yoghurt). The overall KIDMED score ranges from 0 to 12 and are categorized as follows: ≥8 points, good; 4–7 points, average; and ≤3 points, poor MD adherence [45].

All the above questionnaires were fulfilled by qualified personnel (e.g., medical and nursing personnel) and nutritionists and dietitians during face-to-face interviews with the assigned university students.

### 2.4. Statistical Analysis

The continuous variables adopting the normal distribution were analyzed by Student’s *t*-test. Normal distribution was checked by Kolmogorov–Smirnov test. Chi-square was used for categorical variables. Mean value ± Standard Deviation (SD) were applied for expressing continuous variables adopting normal distribution. The categorical variables were expressed as absolute or relative frequencies. Multivariate binary logistic regression analysis was used to evaluate whether the MD compliance of participants may independently be related to sociodemographic, anthropometric, and lifestyle characteristics, including perceived stress and sleep quality, by adjustment for probable confounding factors. All the collected sociodemographic, anthropometric, and lifestyle factors were considered as potential confounding factors in multivariate analysis. The Statistica 10.0 software, Europe was utilized for the statistical analysis (In-former Technologies, Inc., Hamburg, Germany).

## 3. Results

### 3.1. Descriptive Statistics of the Study Population

In Table S1 of the Supplementary Material, the descriptive statistics of the assigned university students are presented. Generally, 5433 university students with a mean age of 21.4 ± 2.5 years old participated in the current survey. In fact, 50.8% of students were female and 79.9% had Greek nationality. Moreover, 61.4% of the participants lived in urban areas and 38.6% in rural areas. Furthermore, 43.6% of the participants exhibited a low financial level, 37.1% had a medium, and merely 19.2% exhibited a high financial level. Concerning living status, 53.8% of the students lived with their family and 46.2% lived alone. Lastly, 33.7% of the students had divorced parents and 39.4% were regular smokers.

In addition, 44.1% of the students performed biomedical studies, like medical, nursing, pharmaceutical, public health or nutritional studies. Additionally, 41.3% of the participants had good academic performance, 37.6% very good academic performance, and only 21.1% exhibited excellent academic performance. Furthermore, 30.8% of the participants were employed. Finally, 15.6% of participants were categorized as overweight and 9.6% as obese.

Regarding the lifestyle parameters of the assigned university students, 40.1% had low physical activity, 33.9% exhibited medium physical activity, and only 26.0% had high physical activity. Besides, 42.6% of the participants were characterized by low levels of perceived stress, 44.0% had moderate levels and 13.4% exhibited high levels of perceived stress. Concerning sleep behavior, 30.6% of the participants had inadequate sleep quality, and 69.4% exhibited adequate sleep quality. Lastly, 47.6% of the participants were characterized by low MD adherence, 34.5% had moderate MD adherence and only 17.9% exhibited high MD adherence.

### 3.2. Association of MD Adherence with Sociodemographic, Anthropometric and Lifestyle Factors

In cross-tabulation, female university students showed significantly higher levels of MD compliance than male students (Table 1, *p* = 0.0001). University students living in rural areas had greater levels of MD compliance than those living in areas (Table 1, *p* < 0.0001). Higher levels of MD compliance were also more often detected in students living with their family as well as with those whose parents were married (Table 1, *p* < 0.0001). University students with higher levels of MD adherence were significantly more often non-smokers compared to those with lower MD adherence levels (Table 1, *p* < 0.0001).

University students performing biomedical sciences and with an excellent academic performance exhibited higher levels of MD compliance (Table 1, *p* = 0.0001 and *p* = 0.0052, respectively). Higher levels of MD compliance were considerably more often noted in non-employee participants compared to employee participants (Table 1, *p* = 0.0287). Likewise, university students with greater levels of MD compliance also exhibited greater physical activity levels (Table 1, *p* = 0.0045). Overweight or obese students considerably showed lower levels of MD compliance than normal weight students (Table 1, *p* < 0.0001). University students presenting greater levels of MD adherence more often had lower levels of perceived stress as well as adequate sleep quality (Table 1, *p* < 0.0001).

### 3.3. Multivariate Binary Logistic Regression Analysis for MD Adherence of the Study Population

**Figure 2:**
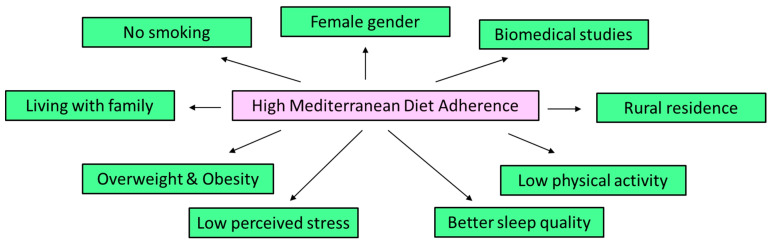
Significant and independent associations of MD adherence with sociodemographic and lifestyle factors including perceived stress and sleep quality.

In multivariate logistic regression analysis MD adherence was independently associated with students’ gender, type of residence, living status, smoking habits, physical activity, BMI, perceived stress and sleep quality (Table 2, *p* < 0.005, Figure 2). More to the point, female students had a 30% higher probability of adopting MD at greater levels compared to male students (Table 2, *p* = 0.0022). Students living in rural regions exhibited a 19% higher likelihood of adhering to MD at greater levels than those living in urban areas (Table 2, *p* = 0.0091). University students living alone showed a 27% higher risk of presenting lower MD adherence than compared to students living with their family (Table 2, *p* = 0.0092). Furthermore, regular smoker university students exhibited a 15% greater likelihood of presenting lower MD adherence than non-smoker university students (Table 2, *p* = 0.0005).

University students studying biomedical sciences had a 22% higher probability of adopting MD at higher levels than those performing other sciences (Table 2, *p* = 0.0188). University students with low MD adherence showed a two-fold higher likelihood of being overweight or obese (Table 2, *p* = 0.007694). University students studying greater levels of physical activity exhibited a 73% higher likelihood of having high MD compliance than those presenting lower levels of physical activity (Table 2, *p* = 0.0081).

Furthermore, university students presenting low MD compliance exhibited a more than two-fold higher risk of developing moderate and higher levels of perceived stress (Table 2, *p* = 0.0037). Additionally, university students presenting low MD adherence had a more than two-fold higher risk of having inadequate sleep quality (Table 2, *p* = 0.0028).

## 4. Discussion

Considering our results, MD adherence among university students was independently linked to various socio-demographic, anthropometric and lifestyle factors. High MD compliance was independently and significantly linked with female gender, rural type of residence, living with family, smoking, biomedicine studies, and being normal weight. Moreover, students who engaged in higher levels of physical activity were 73% more probable to exhibit high MD compliance compared to their less active peers. In addition, those with low MD adherence faced more than twice the risk of experiencing moderate to high levels of perceived stress and inadequate sleep quality, highlighting the potential interplay between dietary habits, mental well-being, and lifestyle factors in this population.

Our study found that low compliance with MD was associated with increased levels of perceived stress among university students, with those demonstrating poor dietary adherence facing more than double the risk of experiencing moderate to high stress levels. This is in accordance with the results of El Mikkawi et al. [46], who also observed that greater MD compliance was related to lower anxiety and stress levels amongst Lebanese university students. Likewise, Antonopoulou et al. [14] reported that students with healthier eating patterns, particularly those closer to the MD, exhibited improved overall mental health, reinforcing the hypothesis that diet quality exerts a key role in psychological well-being among this population. In line with our findings, a cross-sectional survey amongst university students at the age of 18–39 (mean = 20.1 ± 3.0 years) showed that high levels of stress were considerably related to female gender, country of origin, residing off-campus, eating when bored, lack of self-discipline, poor sleep quality, and low levels of life pleasure [47]. In addition, a cross-sectional survey indicated that students presenting greater levels of perceived stress were 2.3 times more likely to have difficulty concentrating on academic work [47]. Accordingly, another cross-sectional survey revealed that greater perceived stress levels was considerably related to less frequent food consumption of fruits and vegetables in males and females [48]. The relationship was more noticeable amongst males compared to females [48]. Similarly, Vélez-Toral et al. [13] supported the positive psychological influence of MD adherence, noting associations with self-esteem and life satisfaction. This study showed that a greater compliance with the MD and the intake of fruits and vegetables were associated with higher psychological adjustment and health perception [13]. Another cross-sectional study also found that students presenting a low compliance with MD exhibited greater levels of stress related to the communication of their own ideas [49].

Unlike studies focusing solely on diet, Abdallah and Gabr [50] emphasized the high prevalence of psychiatric distress among first-year medical students in Egypt, linking it to a range of social, educational, and behavioral factors. Although dietary habits were not directly explored in their work, the substantial levels of stress, depression, and anxiety reported echo the need for multidimensional interventions [50]. A cross-sectional survey performed by Tomás-Gallego et al. [17] found that 29.26% of university students exhibited high compliance to the MD. This study also reported that high MD compliance was related to greater levels of emotional intelligence, as well as lower levels of suicidal ideation, emotional complications, and compulsive internet use [17]. Chacón-Cuberos et al. [49] also reported that students presenting a low compliance with MD exhibited higher levels of stress related to the communication of their own ideas. In another cross-sectional survey, university students showed low compliance with the MD, which had a negative effect on depression, stress, and sleep quality [37]. Our survey contributes to this growing body of evidence by adding a dietary perspective, indicating that promoting MD adherence could be a viable, non-pharmacological approach to reducing stress amongst students, complementing strategies targeting academic, social, and personal stressors.

An additional finding of our survey was that the university students with low adherence to MD were more than twice as likely to experience moderate to high levels of inadequate sleep quality. This aligns with the broader literature, particularly the study by Ünal and Özenoğlu [37], which similarly found that students with poor KIDMED scores had higher stress levels, and in females, significantly poorer sleep quality. Both our and the Ünal and Özenoğlu [37] studies underscore the critical role dietary patterns may play in modulating mental health and sleep amongst university populations. Comparing our data to the findings of Naja et al. [51], stronger compliance with the MD correlates with better sleep quality and healthier circadian preferences (i.e., morningness chronotype). While Naja and colleagues emphasized the chronotype component [51], our results reinforce and expand the understanding of the sleep–nutrition connection by emphasizing the heightened vulnerability of low MD adherents. Our findings are also in line with the systematic review by Fallah et al. [52], which synthesized data from 20 observational studies and concluded that MD adherence may be broadly associated with improved sleep patterns, including aspects like sleep duration and quality. The diversity of tools used in that review like KIDMED, aMED, and MEDAS, reinforces the robustness of the MD–sleep association across methodologies and populations [52].

Another cross-sectional study found a positive relation between healthy diet and sleep quality in Chilean university students [36]. In this study, certain nutritional patterns, like the intake of breakfast and low salt intake and alcohol, were vital to adequate sleep quality in this population group [36]. Ramo-Arbues et al. [38] conducted a cross-sectional survey of 868 university students which found that non-healthy eating was more probably linked to poor sleep quality. The unbalanced consumption of vegetables, fruits, dairy products, lean meats, legumes, sweets and sugary soft drinks was related to poor sleep quality [38]. Another survey aimed to investigate the relationship of levels on the Energy-Adjusted Dietary Inflammatory Index (E-DII) with sleep quality in Lebanese university students [53]. In this survey, university students in the greatest (most proinflammatory) quartile of the E-DII exhibited an elevated risk of presenting inadequate overall sleep quality than those in the lowest quartile [53].

In addition, a recent systematic review and meta-analysis in the general population showed that participants with adequate sleep duration, good-quality sleep, and earlier chronotype exhibited considerably greater odds of high MD compliance than those with inadequate sleep duration, poor-quality sleep, and later chronotype, respectively [54]. A recent scoping review also stated a positive relationship between compliance with MD and improved self-reported sleep quality [55]. A narrative review implied that the vast majority of studies revealed a beneficial association between the MD and sleep duration and/or quality [56]. Nevertheless, most of the above results were achieved in cross-sectional studies [56]. A prospective study also reported that elevated compliance to MD was related to sufficient sleep duration and with several indicators of better sleep quality [57]. Together, the literature and our findings support substantial evidence that MD compliance could be considered as a modifiable lifestyle factor with tangible benefits for sleep hygiene and overall university student well-being.

Our study has clearly indicated that MD adherence may be associated with university students’ well-being related factors, such as academic performance, BMI, physical activity, perceived stress, and sleep quality. In this aspect, a recent cross-sectional study conducted on 936 Cypriot and Greek adults showed that MD adherence was associated with subjective well-being [58]. This study reported that MD compliance was related to higher life satisfaction, a worthwhile life, feeling happy, and self-reported happiness [58]. In Chilean children, higher MD compliance was also associated with greater life satisfaction and subjective well-being [59]. Similar findings were found in another cross-sectional study, which reported a strong positive association between MD compliance and subjective well-being in Portuguese adults [60]. Another study conducted on Spanish adolescents demonstrated that MD compliance, physical activity and weight status were associated with psychological well-being [61]. Notably, adolescents presenting a higher MD compliance showed higher scores in satisfaction of basic psychological needs and satisfaction with life than adolescents with lower MD compliance [61].

### Strengths and Limitations

Our survey design has several strengths, which reinforce our derived results. Firstly, we included a large number of university students from 10 distinct geographical areas of our country, including both urban and rural areas. As a result, our study is adequately representative of the target population group that we initially aimed to examine. Secondly, we used certified questionnaires for evaluating sociodemographic, anthropometric, lifestyle and mental health characteristics of the study population. In this aspect, we aimed to understand if MD adherence is directly associated with perceived stress and sleep quality or if there are other mediating factors that could influence the above relationship. Indeed, even if we included in our multivariate analysis several sociodemographic, anthropometric and lifestyle factors, the strong relationships of adopting MD with perceived stress and sleep quality remained. Thirdly, by performing face-to-face interviews between the assigned students and qualified personnel, we could reduce recall biases, enhancing the validity of the participants responses. Lastly, by including several sociodemographic, anthropometric, and lifestyle factors in our analysis, we obtained an overall overview of whether these factors could be related to MD compliance.

On the other hand, our survey has some limitations. Firstly, our study was performed on university students who exclusively took courses in Greek universities. Thus, our findings cannot be generalized to university students of other countries beyond Greece. Secondly, even if we performed face-to-face interviews to gather our data, these data remain in a self-reported setting and thus recall biases could not entirely disappear. Thirdly, even if we applied multivariate analysis involving several potential confounding factors, there is the possibility of the existence of other confounding factors that we did not take into consideration in the present survey. Lastly, the cross-sectional design of our study cannot permit the derivation of causality effects between MD and perceived stress and sleep quality.

## 5. Conclusions

Our study constitutes one of the first studies that explored whether MD adherence is associated with perceived stress and sleep quality in university students. We found that a healthy diet like the MD could be related to lower incidence of perceived stress and adequate sleep quality in university students. Thus, university students with high MD adherence can obtain a better quality of life throughout their studies, which could also improve their academic performance. However, this survey also highlights the need for future research including longitudinal studies, which may explore whether there is a causality effect between MD compliance and perceived stress and sleep quality of university students. Our findings also emphasize the need to perform educational programs to inform university students about the importance of adopting healthy dietary patterns such as MD to make better their daily quality of life as well as their academic performance. Nutritional interventional programs should also be applied to improve the dietary habits of university students.

## Figures and Tables

**Table 1:** Associations of MD adherence with sociodemographic, anthropometric and lifestyle factors of the study population.

Characteristics (n = 5433)	MD Adherence			
	Low 2589 (47.6%)	Moderate 1873 (34.5%)	High 971 (17.9%)	*p*-Value
**Age (mean ± SD; years)**	21.5 ± 2.5	21.3 ± 2.4	21.2 ± 2.3	*p* = 0.2674
**Gender (n, %)**				*p* = 0.0001
Male	1367 (52.8%)	926 (49.4%)	383 (39.4%)	
Female	1222 (47.2%)	947 (50.6%)	588 (60.6%)	
**Nationality (n, %)**				*p* = 0.0832
Greek	2069 (79.9%)	1578 (84.3%)	696 (71.7%)	
Other	520 (20.1%)	295 (15.7%)	275 (28.3%)	
**Type of residence (n, %)**				*p* < 0.0001
Urban	1811 (70.0%)	1192 (63.6%)	330 (34.0%)	
Rural	778 (30.0%)	680 (36.4%)	641 (66.0%)	
**Family economic status**				*p* = 0.1582
Low	1434 (55.4%)	577 (30.8%)	360 (37.1%)	
Medium	715 (27.6%)	920 (49.1%)	383 (39.4%)	
High	440 (17.0%)	376 (20.1%)	228 (23.5%)	
**Living status (n, %)**				*p* < 0.0001
Living with family	916 (35.4%)	1226 (65.5%)	779 (80.2%)	
Living alone	1673 (64.2%)	647 (34.5%)	192 (19.8%)	
**Parents marital status**				*p* < 0.0001
Not divorced	1510 (58.3%)	1312 (70.0%)	783 (80.6%)	
Divorced	1079 (41.7%)	561 (30.0%)	188 (19.4%)	
**Smoking status**				*p* < 0.0001
Non-smokers	1221 (47.2%)	1323 (70.6%)	748 (77.0%)	
Regular smokers	1368 (52.8%)	550 (29.4%)	223 (23.0%)	
**Type of Studies**				*p* = 0.0001
Biomedical studies	818 (31.6%)	976 (52.1%)	600 (61.8%)	
Other studies	1771 (68.4%)	897 (47.9%)	371 (38.2%)	
**Academic performance**				*p* = 0.0052
Good	1545 (60.0%)	449 (24.0%)	249 (25.6%)	
Very good	841 (32.5%)	1023 (54.6%)	181 (18.7%)	
Excellent	203 (7.8%)	401 (21.4%)	541 (55.7%)	
**Employment status**				*p* = 0.0287
Not employee	1682 (65.0%)	1375 (73.1%)	630 (67.3%)	
Employee	907 (35.0%)	498 (26.6%)	269 (27.7%)	
**Physical activity (n, %)**				*p* = 0.0045
Low	2038 (78.7%)	346 (18.5%)	183 (18.8%)	
Medium	397 (15.3%)	1318 (70.4%)	124 (13.8%)	
High	154 (6.0%)	209 (11.2%)	664 (68.4%)	
**BMI (n, %)**				*p* < 0.0001
Normal weight	2033 (78.5%)	1416 (75.6%)	619 (63.8%)	
Overweight	337 (13.0%)	325 (17.3%)	180 (18.5%)	
Obese	219 (8.5%)	132 (7.1%)	172 (17.7%)	
**Perceived stress (n, %)**				*p* < 0.0001
Low	1444 (57.4%)	629 (33.6%)	196 (20.2%)	
Moderate	706 (27.3%)	1012 (54.0%)	673 (69.3%)	
High	395 (15.3%)	232 (12.4%)	102 (10.5%)	
**Sleep Quality (n, %)**				*p* < 0.0001
Adequate	1530 (59.1%)	1471 (78.5%)	768 (79.1%)	
Inadequate	1059 (40.9%)	402 (21.5%)	203 (20.9%)	

**Table 2:** Multivariate analysis for MD adherence of study population.

Characteristics	MD Adherence (Low vs. Moderate and High)	
	OR * (95% CI **)	*p*-Value
**Age** (Over/Below mean value)	1.12 (0.21–2.23)	*p* = 0.4918
**Gender** (Male/Female)	0.70 (0.59–0.95)	*p* = 0.0022
**Nationality** (Greek/Other)	1.12 (0.32–1.91)	*p* = 0.5032
**Type of residence** (Urban/Rural)	0.81 (0.62–1.01)	*p* = 0.0091
**Family economic status** (Low/Medium and High)	0.85 (0.31–1.43)	*p* = 0.2019
**Living status **(Living alone/Living with family)	0.73 (0.62–0.90)	*p* = 0.0092
**Parents marital status** (Divorced/No divorced)	0.98 (0.29–1.78)	*p* = 0.3419
**Smoking status** (Regular smokers/No smokers)	1.15 (0.93–1.26)	*p* = 0.0005
**Type of Studies** (Biomedical studies/Other studies)	0.78 (0.59–0.97)	*p* = 0.0188
**Academic performance** (Good/Very good and Excellent)	0.88 (0.31–1.39)	*p* = 0.0809
**Employment status** (Employee/No employee)	1.13 (0.56–1.98)	*p* = 0.4019
**BMI** (Overweight and obese/Normal weight)	2.07 (1.92–2.25)	*p* = 0.0076
**Physical activity** (Low/Medium and High)	1.73 (1.58–1.97)	*p* = 0.0081
**Perceived stress** (Moderate and High/Low)	2.21 (1.99–2.47)	*p* = 0.0037
**Sleep Quality** (Inadequate/Adequate)	2.32 (2.09–2.54)	*p* = 0.0028

* OR: Odds Ratio, and CI **: Confidence Interval.

## Data Availability

The data of this study are available on request to the corresponding author.

## Supplementary Materials

The following supporting information can be downloaded at: https://www.mdpi.com/article/10.3390/nu17132174/s1, Table S1. Descriptive statistics of the study population.
