# Supplementary material for: Mediterranean Diet Compliance Is Related with Lower Prevalence of Perceived Stress and Poor Sleep Quality in University Students: A Cross-Sectional Study in Greece
Source: Nutrients. 2025 Jun 30;17(13):2174. doi: 10.3390/nu17132174 (PMC12252162; doi:10.3390/nu17132174)
Supplement: Supplementary file 1 [file nutrients-17-02174-s001.zip › nutrients-3726532-supplementary.pdf]

Table S1. Descriptive statistics of the study population.

Characteristics (n=5433)	Descriptive statistics
**Age (mean±SD; years)**	21.4±2.5
**Gender (n, %)**	
Male	2676 (49.2%)
Female	2757 (50.8%)
**Nationality (n, %)**	
Greek	4343 (79.9%)
Other	1090 (10.1%)
**Type of residence (n, %)**	
Urban	3334 (61.4%)
Rural	2099 (38.6%)
**Family economic status**	
Low	2371 (43.6%)
Medium	2018 (37.1%)

High	1044 (19.2%)
**Living status (n, %)**	
Living with family	2921 (53.8%)
Living alone	2512 (46.2%)
**Parents marital status**	
No divorced	3605 (66.3%)
Divorced	1828 (33.7%)
**Smoking status**	
No smokers	3292 (60.6%)
Smokers	2141 (39.4%)
**Type of Studies**	
Biomedical studies	2394 (44.1%)
Other studies	3039 (55.9%)
**Academic performance**	
Good	2243 (41.3%)

Very good	2045 (37.6%)
Excellent	1145 (21.1%)
**Employment status**	
Employee	1674 (30.8%)
No employee	3759 (69.2%)
**Physical activity (n, %)**	
Low	2181 (40.1%)
Medium	1839 (33.9%)
High	1413 (26.0%)
**BMI (n, %)**	
Normal weight	4062 (74.8%)
Overweight	846 (15.6%)
Obese	525 (9.6%)
**Perceived stress (n, %)**	
Low	2313 (42.6%)

Moderate	2391 (44.0%)
High	729 (13.4%)
**Sleep Quality (n, %)**	
Adequate	3769 (69.4%)
Inadequate	1664 (30.6%)
**Mediterranean Diet Adherence (n, %)**	
Low	2589 (47.6%)
Moderate	1873 (34.5%)
High	971 (17.9%)
